# Missed opportunities to address common mental disorders and risky alcohol use among people living with HIV in Zomba, Malawi: A cross sectional clinic survey

**DOI:** 10.1371/journal.pone.0278160

**Published:** 2023-02-06

**Authors:** Harry Henry Kawiya, Thandi Davies, Crick Lund, Katherine Sorsdahl

**Affiliations:** 1 Clinical Department, Ministry of Health, Zomba Mental Hospital, Zomba, Malawi; 2 Department of Psychiatry & Mental Health, Alan J. Flisher Centre for Public Mental Health, University of Cape Town, Cape Town, South Africa; 3 Health Service and Population Research Department, Centre for Global Mental Health, Institute of Psychiatry, Psychology and Neuroscience, King’s Global Health Institute, King’s College London, London, United Kingdom; Duke University Medical Center: Duke University Hospital, UNITED STATES

## Abstract

Common mental disorders (CMDs) and risky alcohol use are highly prevalent among people living with HIV, yet many do not receive treatment for these mental health problems. In Malawi, despite a mental health policy aiming to include mental healthcare into primary health care, many clients with HIV go unscreened and untreated for mental illnesses, indicating missed opportunities to offer mental health care to people living with HIV. The aim of this study was to determine the numbers and types of missed opportunities for screening and treatment of CMDs and risky alcohol use amongst a sample of people living with HIV attending anti-retroviral (ART) clinics in Zomba Malawi. A descriptive cross-sectional clinic survey was used, at three ART clinics in the Zomba district. Random sampling was conducted for all clients attending their ART clinics on specific days. The study surveyed 382 participants living with HIV. Of these participants, the majority were women (N = 247, 64.7%), and 87 (22.8%) screened positive for CMDs and/or alcohol misuse using the self-reporting questionnaire 20 (SRQ-20) and alcohol use disorder identification test (AUDIT). Of these, only 47 (54%) had been screened by health workers for CMDs or risky alcohol use in the past 12 months, and 66 (76%) wanted to receive treatment. Of the total sample of 382 participants, only 92 (24%) and 89 (23%) had been screened for CMDs or risky alcohol use by health workers. Failures by clinical officers and nurses to screen or treat CMDs and risky alcohol use in ART clinics represent missed opportunities to address the mental health of people living with HIV. Providing psychoeducation for staff, guidelines for screening and managing CMDs and alcohol use, increasing human resources, and accelerating implementation of the mental health policy in Malawi may be a few ways of improving mental health service provision at ART clinics in Malawi.

## Introduction

Common mental disorders (CMDs), which encompass mood and anxiety disorders, and risky alcohol use, are highly prevalent among people living with HIV [1]. Various systematic reviews and meta-analyses have been conducted across different regions of Africa, and all reported high rates of common mental disorders (CMDs), among people living with HIV [2]. Prevalence of depression was found to be 38% in East Africa [3], 15.3%, in Sub-Saharan Africa [4], and prevalence of CMDs in HIV positive clients in Ethiopia was reported to be 28.8% [5]. Another systematic review and meta-analysis among people living with HIV in Africa reported prevalence of alcohol use disorder at 16.7% [6].

Despite this high prevalence, studies have found substantial evidence that many people who suffer from CMDs and engage in risky alcohol use, including those living with HIV, do not receive treatment for their mental health problems. This is referred to as the treatment gap: defined as the discrepancy between those that have mental health problems and those that have received treatment for them. A cohort study conducted in South Africa found a large treatment gap among people with mental disorders that were accessing HIV care services both in private and public services. The percentage of people not receiving treatment was found to be 40.5% and 95.5% for private and primary public sector clinics respectively [7]. Globally, in 2004 the treatment gap for those who had met criteria for alcohol abuse and dependence was 78% (with percentages ranging between 49.4% in Israel and 96% in Mexico City [8].

Although there are several contributors to this large treatment gap, one of the primary contributors is the shortage of trained mental healthcare workers [9]. In order to address this problem, the strategy of integrating mental healthcare into general primary care settings through task-sharing has been widely advocated [10]. Task-sharing can be defined as providing an opportunity for non-mental healthcare workers to perform the tasks of a specialist mental healthcare worker through appropriate training and supervision [11]. Many countries have employed the World Health Organisation (WHO) mental health Gap Action Programme Intervention Guide (mhGAP-IG) to assist them in integrating mental health into general primary care services [12].

Malawi is one such country that has adopted the mhGAP-IG to guide mental health policy and strategic plans for mental healthcare delivery. Despite this, poor detection rates of mental health problems among healthcare workers have been reported in some areas of Malawi. For example, one study conducted in a primary health care (PHC) clinic found that 20.1% of participants met the criteria for probable CMD, yet no clients were identified by PHC staff [13]. In spite of some local studies reporting poor detection or screening, two local studies reported an improvement in knowledge and detection rate of mental health problems after training. For example, one study reported significant improvement in knowledge on a 20-point mhGAP knowledge questionnaire, from mean score of 11.8 prior mhGAP-IG training to 15.1 soon after training [14]. The other study reported a significant improvement on rate of detection of mental health problems by primary healthcare workers, from 0% at baseline to 9.2% post training [15]. If CMDs and risky alcohol use are not identified by primary healthcare providers, this constitutes a missed opportunity to provide services. Additionally, if needs for additional services are identified and addressed on the same clinic visit, higher coverage and more cost-effective service delivery can be achieved.

The definition of missed opportunities utilized in most studies has centered on failure to receive services and address client needs despite meeting the criteria for mental health problems [16]. A study investigating missed opportunities to screen for risky alcohol use in women’s health settings in Virginia USA defined a missed opportunity as a failure of a healthcare provider to ask or screen for alcohol use and offer a brief intervention [17]. In this study, of the women who reported drinking alcohol, 30% were not asked about their alcohol use and 82% who were classified as risky drinkers did not receive a brief intervention from the healthcare provider [17]. In addition, clients often indicate a willingness to receive a mental health intervention, even if they have not been screened for mental health problems. For example, a South African study found that 46% of clients who were waiting for their medical visit indicated willingness to receive a mental health intervention for depression, substance use and/or suicide [16].

In Malawi, although missed opportunities have not been investigated specifically using the definitions described by Hettema J. *et al*. and Sorsdahl K. *et al*. [16, 17], there is evidence that patients who attend general healthcare facilities with probable CMDs go undetected and untreated [13]. This study aimed to explore this further by investigating the missed opportunities to address CMDs or risky alcohol use among people living with HIV during routine antiretroviral therapy (ART) clinic visits in Zomba, Malawi. In this study a missed opportunity was defined as occurring when, during any of their ART visits in the last twelve months, a Clinical Officer (CO) or nurse failed to inquire about or screen for CMDs or alcohol use in clients who met clinical criteria for these conditions, and furthermore when they failed to refer the client for advice or treatment.

## Materials and methods

### Setting

The study was conducted in the Zomba district of Malawi, where there are a total of 37 health facilities. Fourteen of these are public primary healthcare facilities and two are tertiary hospitals: Zomba Central Hospital (ZCH), which is a referral hospital for physical diseases, and Zomba Mental Hospital (ZMH). All 37 health facilities offer ART. A convenience sample of three ART clinics, namely Tisungane, Matawale and Domasi ART clinics, were selected as the setting for this study, based on the clinics’ high average attendance rates by people living with HIV. Tisungane ART clinic is operated under Zomba Central Hospital. Tisungane and Matawale ART clinics are located within Zomba City while Domasi ART clinic is on the outskirts of Zomba City, and mainly provides care to rural clients. There are a number of monitoring services which are offered routinely at ART clinics, such as for HIV-related diseases, ART failure, drug side effects and nutritional status. However, despite having adopted the mhGAP to guide mental health, screening for mental health problems (probable CMDs and risky alcohol use) is not done routinely during ART clinic visits.

### Sample and procedure

During days of data collection, a list of all the clients booked for the day was drawn from the clinic. From this list, random sampling using a computer was conducted for all clients, to select potential participants. Selected participants were then informed that a research study was being carried out to investigate the mental health of people living with HIV receiving care at each ART clinic, and were invited to participate in the study.

The inclusion criteria for individuals to participate in the study were: (1) Documented HIV positive test result, (2) age 18 or older seeking or receiving treatment in one of the three HIV treatment centres involved in this study. The exclusion criteria were: (1) clients that had a serious medical condition requiring immediate attention by clinician, or (2) had been diagnosed with a severe mental illness, or and (3) did not consent to participate in the study.

No participant refused to take part in the study. Participants were interviewed immediately after their appointment with the primary healthcare worker. They were taken to a private room where the study was explained in more detail and the process of informed consent was completed. The interview took approximately 20 minutes and research assistants conducting the interviews were experienced Psychiatric Clinical Officers who were employed for the purpose of the study, and were not involved in routine clinical care in these facilities. Data was collected for four weeks.

The study was approved by the University of Cape Town Human Research Ethics Committee (HREC) in South Africa and the National Health Sciences Research Committee in Malawi. Approval to conduct the study was obtained from the Director of Health and Social Services (DHSS) of Zomba District and the Director of Zomba Central Hospital. Thereafter permission was obtained from the Health Facility Managers of Domasi, Matawale and Tisungane ART clinics. Informed written consent was obtained from the study participants before commencement of administering the questionnaire. Participants were given adequate information regarding the aims and benefit of the study and then each was free to give an informed consent before answering the questionnaire. Information sheets were provided to the participants and these were available in both English and Chichewa; participants were given a choice whether to take the English or Chichewa version. For the participants who were unable to read, information was read to them. Furthermore, those that could not write were asked mark consent using their thumbprints.

Confidentiality of the participants was upheld such that their responses and identity were not revealed to anybody else. Questionnaires were administered in private rooms at the health facilities. The completed questionnaires were kept in a lockable room accessible only to the researcher. Research assistants were trained on confidentiality and research ethics in general.

### Measures

Demographic data collected included age, education status, marital status, residential area, and employment, months living with HIV, monthly income, monthly expenditure, number of clinic visits to collect ART and number of clinic visits of HIV related complaints.

The Self-Reporting Questionnaire (SRQ-20) was used to identify cases of probable CMDs. It has 20 items and was developed by the WHO to screen for probable CMDs in primary health care and community level healthcare in developing countries. It has been translated to Chichewa and validated in Malawi using a recommended cut off score of eight or above to indicate probable CMDs, with a sensitivity of 71% and a specificity of 92% [18]. The Alcohol Use Disorder Identification Test (AUDIT) was used to identify cases of risky alcohol use, using a cut off score of eight or more. This screening tool has 10 questions. It has been found to provide an accurate measure of risky alcohol use across gender, age, and cultures [19]. The present study adopted a version which was being validated in Malawi by B Mwagomba through the University of St Andrews, Scotland.

To collect data on missed opportunities, participants were asked whether in their visits to the clinic in the past 12 months: (1) a Clinical Officer (CO healthcare worker who undergoes three years of training for clinical medicine)) or nurse had asked them about their feelings, such as feeling unhappy, feeling weak, sleeping poorly, eating poorly, emotional or worrying (to screen for probable CMDs), or about their alcohol use; (2) whether advice or treatment for mental health problems was recommended; (3) whether they were referred to a mental health specialist; and (4) whether they would have liked to have received advice or treatment regarding mental health problems or alcohol use.

### Data analysis

Data was analyzed using SPSS Version 25. Descriptive statistics (means, standard deviations and frequency analysis) were presented. Determination of CMDs and risky alcohol use was done using the SRQ and AUDIT cut off scores, and the variables were dichotomized for presence or absence of CMDs and risky alcohol use. Unadjusted and adjusted associations between clinical, social and demographic characteristics (independent variables) and missed opportunities (dependent variables) were explored through multiple logistic regression. The results of the regression models were reported as odds ratios (ORs) with 95% confidence intervals [20]. Significance was set at p<0.05.

## Results

### Socio-demographics

The study included 382 participants in total, with an average age of 41 years (SD 10.4). Of these participants, more than half were women (N = 247, 65%) Two-thirds of the participants were married (N = 253, 66%), 55% had completed primary school education (N = 210), and 65% resided in rural communities (N = 247). Most (75%) of participants earned their income through employment and small-scale business. The mean monthly income of all participants was $52.6 (SD 64.6) which was equivalent to MK38, 924 (Malawian Kwacha), with monthly income ranging between $0.3 to $402.1. The monthly mean expenditure of participants was $44.1 (SD 57.5) per month. The study participants had been living with HIV between 0–276 months with an average of 85 months (SD 59.0). The number of clinic visits to collect antiretroviral therapy in the past 12 months ranged from 1–14 clinic visits, with a mean of 5.1 visits (SD 2.3). Clinic visits for HIV-related complaints in the past 12 months ranged from 1–12, with a mean of 4.5 visits (SD 1.9). Table 1 presents this data.

**Table 1:** Socio-demographic characteristics of the participants.

	Total (N = 382)	Males (N = 135)	Females (N = 247)	
	**N, %**	**N, %**	**N, %**	**p-value**
**Age** (m, sd)	41.4, 10.4	44.3, 10.9	39.9, 9.6	<0.001*
**Education**				0.14
Completed primary school	210 (55)	81 (60.0)	129 (52.2)	
No primary school primary	172 (45)	54 (40.0)	118 (47.8)	
**Marital status**				<0.001*
Married	253 (66.2)	113(83.7)	140(56.7)	
Single	129 (33.8)	22 (16.3)	107 (43.3)	
**Residential area**				0.95
Urban	135 (35.3)	48 (35.6)	87 (35.2)	
Rural	247 (64.7)	87 (64.4)	160 (64.8)	
**Employment**				<0.001*
Employed	283 (74.1)	115 (85.2)	168 (68.0)	
Not employed	99 (25.9)	20 (14.8)	79 (32.0)	
**Months living with HIV (m, sd)**	85.1, 59.0	84.2, 59.9	85.6, 58.6	0.834
**Monthly income in $ (m, sd)**	52.6, 64.6	61.7, 67.1	47.7, 62.9	0.04*
**Monthly expenditure in $ (m, sd)**	44.1, 57.5	56.3, 61.7	37.4, 54.0	0.002*
**No. of clinic visits- collect ART (m, sd)**	5.1, 2.3	5.3, 2.4	5.0, 2.3	0.27
**No. of clinic visits-HIV complaints (m, sd)**	4.5, 1.9	4.7, 2.0	4.4, 1.8	0.13

### Mental health problems

Using the SRQ-20, 77 study participants (20.2%) screened positive for CMDs. More female participants screened positive for CMDs (63/77, 81.8%). Risky alcohol use (on the AUDIT) among people living with HIV was reported by 16 participants (4.2%) and was higher among male participants (11/16, 68.8%). In total, 87 (22.8%) participants screened positive for probable mental health problems.

### Missed opportunities

With regards to ‘missed opportunities’, 92 (24%) and 89 (23%) participants had been asked by COs or nurses in the last 12 months whether they struggled with their feelings (probable CMDs), or alcohol use, respectively. Some nurses only asked about CMDs, and not alcohol use, and vice versa. Among those who were asked about probable CMDs and risky alcohol use, 47 participants (approximately 24%) screened positive for CMDs or risky alcohol use in this study.

Of those who had been asked about mental health, only 11 (12.6%) and 15 (17.2%) participants went on to receive advice or treatment from COs or nurses for probable CMDs and risky alcohol use, respectively. From the total sample, participants who wanted advice for their feelings and alcohol use were 316 (82.7%) and 190 (49.7%) respectively. Among these participants, 79 (20.7%) screened positive in the study. Table 2 presents this data.

**Table 2:** Proportion of respondents who screened positive, who were asked, who wanted advice or treatment and who received advice or treatment for mental health problems.

	Total sample	Male	Female
	N = 382	N = 135	N = 247
	N (%)	N (%)	N (%)
**Screened at risk by study**			
CMDs only	77 (20.2)	14 (3.7)	63 (16.5)
Risky alcohol use only	16 (4.2)	11 (2.9)	5 (1.3)
Probable mental health problems	87 (22.8)	23 (17.0)	64 (25.9)
**Participants who were asked/ screened by nurses**			
Probable CMDs only	92 (24.1)	43 (11.3)	49 (12.8)
Risky alcohol use only	89 (23.3)	33 (8.6)	56 (14.7)
Proportion of these screened positive by study	47 (28.5)	18 (25.7)	29 (30.5)
**Participants who wanted advice for mental health problems**			
Probable CMDs only	316 (82.7)	115 (30.1)	201 (52.6)
Risky alcohol use only	190 (49.7)	74 (19.4)	116 (30.4)
Proportion of these screened positive by study	79 (22.3)	22 (16.7)	57 (25.7)

To examine missed opportunities further, we further examined data for the 87 participants who screened positive for common mental disorders (CMDs) and risky alcohol use in the current study (and not by nurses in the past 12 months). Four definitions of missed opportunities were operationalized for this, and **Table 3** provides definitions and proportion of participants for each ‘missed opportunity’.

**Table 3:** Definitions of missed opportunities (N = 87).

Definitions	N (%)
Missed opportunity for screening definition # 1. Screened positive by study but not screened by nurse.	40 (46.0)
Missed opportunity for screening definition #2. Screened positive by study, not screened by nurse, but wanted advice or treatment.	35 (40.2)
Missed opportunity for treatment definition #3. Screened positive by study, may or may not have been screened by nurses, but did not receive advice or treatment.	87 (100.0)
Missed opportunity for treatment definition #4/ Screened positive by study, may or may not have been screened by nurses, did not receive advice or treatment, but wanted treatment.	66 (75.9)

### Missed opportunities for mental health identification/screening

For missed opportunity 1, a respondent had to have screened positive for CMDs and risky alcohol use in the current study (N = 87), but a nurse had not inquired about their CMDs and risky alcohol use in any of their visits to the clinic in the past 12 months. This study found that 46% (N = 40) fulfilled missed opportunity 1. After adjusting for demographic variables, only female gender was significantly associated with missed opportunity 1. **See Table 4** for these associations.

**Table 4:** Unadjusted and adjusted associations between socio-demographics and missed opportunity #1.

	Not screened by nurse N (%)	Screened by nurse N (%)	Unadjusted OR	Adjusted OR
	**N = 40 (46.0)**	**N = 47 (54.0)**	**(95% CI)**	**(95% CI)**
**Gender**				
Male	5 (12.5)	18 (38.3)	1.00	1.00
Female	35 (87.5)	29 (61.7)	4.35 (1.44–13.14) *	4.44(1.43–13.74) *
**Age m (sd)**	37.68 (8.58)	41.47 (9.51)	0.95 (0.91–1.00)	0.97 (0.93–1.00)
**Marital status**				
Not married	15 (37.5)	22 (46.8)	1.00	
Married	25 (62.5)	25 (53.2)	1.47 (0.62–3.46)	
**Residential area**				
Urban	11 (27.5)	19 (38.3)	1.00	
Rural	19 (72.5)	28 (61.7)	1.79 (0.72–4.43)	
**Employment**				
Not employed	15 (37.5)	10 (21.3)	1.00	
Employed	25 (62.5)	37 (78.7)	0.45 (0.17–1.16)	
**Education**				
Not educated	19 (47.5)	18 (40.4)	1.00	
Educated	21 (52.5)	29 (59.6)	0.69 (0.29–1.61)	
**Months living with HIV (m, sd)**	95.25 (61.94)	80.77 (65.14)	1.01 (0.99–1.01)	
**Monthly income in $ (m, sd)**	54.24 (75.70)	67.00 (76.30)	1.01 (1.00–100)	
**Monthly expenditure in $ (m, sd)**	34.82 (43.82)	54.96 (60.22)	0.99 (0.98–1.01)	
**No. of clinic visits: ART collection (m, sd)**	4.98 (2.24)	6.02 (4.83)	0.91 (0.79–1.06)	
**No. of clinic visits -HIV complaints (m, sd)**	4.30 (1.95)	4.70 (2.25)	0.91 (0.74–1.12)	

For missed opportunity 2, a study participant who screened positive had to have been undetected for CMDs and risky alcohol use by nurses in any of his or her visits to the clinic in the past 12 months, and in addition, s/he wanted to receive advice or treatment about his or her CMDs and risky alcohol use. This study found that 35 (40.2%) of participants met missed opportunity 2.

Female gender (OR = 4.46, 95% CI 1.37–14.59) and young age (OR = 0.94, 95% CI 0.89–0.99) were significant in the unadjusted associations for Missed Opportunity 2. Employment (OR = 0.40, 95% CI 0.16–1.04) and monthly expenditure (OR = 0.99, 95% CI 0.98–1.00) were close to significance. These were included in the multiple logistic regression analysis. After adjusting for these variables in the model, female gender (OR = 3.94, 95% CI 1.10–14.09), young age (OR = 0.96, 95% CI 0.92–1.00), employment (OR = 0.30, 95% CI 0.10–0.91) and monthly expenditure (OR = 0.98, 95% CI 0.96–1.00) were significantly associated with missed opportunity definition 2. **See Table 5**.

**Table 5:** Unadjusted and adjusted associations between socio-demographics and missed opportunity #2.

	Not screened/ didn’t want treatment	Not screened/ wanted treatment	Unadjusted OR (95% CI)	Adjusted OR (95% CI)
Variable	**N = 52**	**N = 35**		
**Gender**				
Male	19 (36.5)	4 (11.4)	1.00	1.00
**Female**	33 (63.5)	31 (88.6)	4.46 (1.37–14.59) *	3.94 (1.10–14.09) *
**Age m (sd)**	41.73 (9.25)	42.22 (10.92)	0.94 (0.89–0.99) *	0.92 (0.87–0.98) *
**Marital status**				
Not married	25 (48.1)	51 (30.2)	1.00	
Married	27 (51.9)	118 (69.8)	1.78 (0.73–4.3)	
**Residential area**				
Urban	20 (42.3)	9 (25.7)	1.00	
Rural	31 (57.7)	26 (74.3)	1.96 (0.77–5.00)	
**Education**				
Not educated	22 (42.9)	15 (42.9)	1.00	
Educated	30 (57.1)	20 (57.1)	0.98 (0.41–2.33)	
**Employment**				
Not employed	11 (21.2)	14 (40.0)	1.00	1.00
Employed	41 (78.8)	21 (60.0)	0.40 (0.16–1.04) *	0.30 (0.10–0.91) *
**Months living with HIV (m, sd)**	83.37 (62.93)	87.07 (58.48)	1.01 (0.99–1.01)	
**Monthly income in $ (m, sd)**	70.80 (77.84)	46.79 (71.48)	0.99 (0.99–1.001)	
**Monthly expenditure in $ (m, sd)**	55.51 (58.58)	31.12 (43.04)	0.99 (0.98–1.00)	0.98 (0.96–1.00) *
**No. of clinic visits to collect ART in past 12 months (m, sd)**	5.90 (4.64)	5.00 (2.30)	0.93 (0.80–1.07)	
**No. of clinic visits related HIV complaints in the 12 months (m, sd)**	4.63 (2.15)	4.34 (2.07)	0.93 (0.75–1.16)	

* = p<0.05.

### Missed opportunities for mental health advice/treatment

For missed opportunity 3, a respondent had to have screened positive for CMDs and risky alcohol use in the current study (N = 87), may or may not have been screened by a CO or nurse, but did not receive advice or treatment for these problems. All 87 participants had not been given advice or treatment and thus met the criteria for missed opportunity 3. Given that all 87 met this criterion, we were unable to develop logistic regression models.

For missed opportunity 4, a study positive participant may or may not have been screened by a CO or nurse, did not receive advice or treatment for these problems, but wanted to receive advice or treatment. Approximately two thirds (N = 66, 75.9%) of participants fulfilled missed opportunity 4. Unadjusted associations between socio-demographic and clinical related variables and missed opportunity 4 are reported in **Table 6.** There were no significant associations.

**Table 6:** Unadjusted associations between socio-demographics and missed opportunity #4.

	Study positive/wanted treatment/wasn’t given treatment, %	Study positive/did not want treatment, %	Unadjusted OR (95% CI)
Variable	**N = 66 (75.9)**	**N = 21 (24.1)**	
**Gender**			
Male	15 (22.7)	8 (38.1)	1.00
Female	51 (77.3)	13 (61.9)	2.09(0.73–5.99)
**Age m (sd)**	39.80 (9.48)	39.71(8.71)	1.01 (0.95–1.06)
**Marital status**			
Married	29 (43.9)	(44.8)	1.00
Not married	37 (56.1)	37 (55.2)	0.79 (0.29–2.15)
**Residential area**			
Urban	20 (30.3)	10 (47.6)	1.00
Rural	46 (69.7)	11 (52.4)	2.09 (0.77–5.71)
**Education**			
Educated	28 (42.4)	9 (42.9)	1.00
Not educated	38 (57.6)	12 (57.1)	1.02 (0.38–2.75)
**Employment**			
Employed	20 (30.3)	5 (23.8)	1.00
Not employed	46 (69.7)	16 (76.2)	0.72 (0.23–2.23)
**Months living with HIV (m, sd)**	85.91 (60.99)	94.43 (72.73)	0.99 (0.98–1.01)
**Monthly income in $ (m, sd)**	53.40 (65.97)	85.47 (98.87)	1.00 (1.00–1.00)
**Monthly expenditure in $ (m, sd)**	42.05 (47.66)	57.16 (70.42)	0.99 (0.99–1.01)
**No. of clinic visits in past 12 months (m, sd)**	5.85 (4.29)	4.57 (1.86)	1.18 (0.94–1.48)
**No. of HIV related clinic visits in the 12 months (m, sd)**	4.70 (2.32)	3.95 (1.12)	1.24 (0.91–1.70)

## Discussion

This study is, to the best of our knowledge, the first to investigate missed opportunities to address CMDs and risky alcohol use among people living with HIV in Zomba, Malawi. Notably, the study found that there was a prevalence of 20.2% of CMDs in clients attending ART clinics. Risky alcohol use was far less prevalent in this sample (4.2%).

The prevalence of CMDs found in this study was similar to another study conducted in general primary care in Malawi, in the Zomba district, where prevalence of CMDs were found to be 20.1% [13]. However, the findings in this current study were low compared to the closest available data, from a neighbouring country, Zimbabwe, where reported prevalence of CMDs was almost three times higher than the current study. One Zimbabwean study found the prevalence of CMDs among clients with HIV to be 67.9% [21], and another found prevalence of comorbid probable post-traumatic stress disorder and CMDs to be 65% among people living with HIV [22].

The prevalence of risky alcohol use was significantly lower than previous studies that have reported on risky alcohol use among people living with HIV in other African countries. For example, a systematic review examining the mental health of people living with HIV in Africa found the prevalence of alcohol abuse and dependence to be between 7% and 16% [2]. Possible reasons for the low rates are that generally alcohol consumption is low in Malawi compared to other countries [23, 24]. Furthermore, participants might have under-reported alcohol consumption, due to anticipated stigma or concerns regarding the care they are receiving at the clinic.

### Factors associated with missed opportunities for screening

The present study found some socio-demographic factors that were significantly associated with missed opportunities. Missed opportunity definition #1 was significantly associated with female gender while missed opportunity definition #2 was significantly associated with female gender and young age. Female participants were more likely to meet criteria for both missed opportunities #1 and #2. Women are more likely to experience symptoms of probable CMDs than men. Given that most participants in the present study were female, it is not surprising that many women with symptoms of CMDs were identified at the clinics compared to males.

After adjusting for these variables in the model, female gender (OR = 3.94, 95% CI 1.10–14.09), age (OR = 0.96, 95% CI 0.92–1.00), employment (OR = 0.30, 95% CI 0.10–0.91) and monthly expenditure (OR = 0.98, 95% CI 0.96–1.00) were significantly associated with missed opportunity definition #2. Young participants were more likely to meet criteria for missed opportunity definition #2 compared to older participants. A possible reason for this is that health workers may have more concerns for the health of older than younger participants and therefore are more likely to ask about their health than they would with younger clients. Participants who were employed were less likely to meet criteria for missed opportunity definition #2. This may be because clinical officers and nurses might have more respect for people who are employed due to their higher quality of life. In the same vein, participants who were spending less were less likely to meet criteria for missed opportunity definition #2. There was also a discrepancy in the findings between income and expenditure. This may be due to income being under-reported in the survey, and that the questionnaire only asked the participants to declare their total income and total expenditure.

### Missed opportunities

Several important findings relating to missed opportunities were noted in this study. First, screening or enquiry about probable CMDs and alcohol use by clinical officers (CO) or nurses was low, at 92 and 89 out of the total sample of 382 (approximately 24%). Of the screened positive sample in the study (N = 87), only 47 (54%) had been asked by nurses about their mental health (meeting the criteria for missed opportunity 1). Those health workers that did enquire about mental health may have been influenced by their training: in Malawi, all medical and nursing students attend a practical attachment for mental health and psychiatry for two-to-six weeks at the psychiatric hospital.

A study from central Malawi demonstrated that screening by health care workers is possible: the study piloted the integration of depression management into HIV primary care and found that HIV counsellors screened 88.3% and 93.2% of newly diagnosed seropositive clients for depression at two study sites [25]. However, they note that there were still challenges in increasing the capacity of counsellors to fully diagnose and manage depression at PHC settings.

Second, regarding advice or treatment, although COs and nurses were asking some clients about mental health problems, the rate of provision of advice or treatment was poor. These findings show that nurses working in ART clinics were not giving advice or treatment to clients despite enquiring about probable CMDs and risky alcohol use. This study found that 100% of participants who had screened positive by the study screening tools did not receive advice or treatment from COs or nurses. More than three-quarters of these participants wanted to receive advice or treatment for these problems (75.9%).

In addition, most participants in the total study sample (N = 382), including those who did not screen positive for mental health problems, stated that they wanted to receive advice or treatment for CMDs and alcohol use (82.7% and 49.7%). This indicates an unmet need amongst this population. Studies have found that clients want to receive advice or treatment for mental health problems. For example, a study conducted in South Africa that examined patient preferences for the integration of mental health counseling and chronic disease care found that participants accepted screening and counselling for depression and alcohol use within their care visits to clinic [26]. Another South African study similarly found that 46% of participants who were waiting for their medical visit indicated willingness to receive a mental health intervention for depression, substance use and suicide, but did not receive advice [16].

### Possible reasons for low provision of treatment

However, low provision of advice or treatment to clients by nurses is in keeping with traditional practice in Malawi, whereby mental health treatment or advice-giving is viewed as a specialized field of medicine. One local study that investigated nurses’ knowledge and skills in providing mental health care at general primary healthcare clinics found that 58.8% of nurse participants did not know how to treat mental health problems [27].

Another potential reason for low provision of treatment or advice giving could be that non-specialised mental healthcare workers may feel that they need more time to treat mental health problems than they have [28]. Furthermore, issues of mental health treatment are not well articulated in the Malawi clinical guidelines for management of HIV [29]. It is possible that nurses feel that mental health services are not part of their work since it is not clearly articulated in HIV management guidelines.

A shortage of human resources in clinics might be another reason for the lack of advice and treatment provided to clients by clinical officers and nurses. Several studies conducted on human resources have pointed to a shortage of healthcare workers, for example, a study examining the mental health workforce in LMICs found that the 58 LMICs sampled would need to recruit 239,000 full-time professionals to address current shortages in mental healthcare staffing [30]. This shortage is worse in Malawi, where UNAIDS has found that Malawi has one of the most severe health workforce crises in Africa. The physician to population ratio is currently at 2:100,000 [31], while the ratio of nurse to population is the second lowest in Africa, at 28:100,000.

The last reason that might have contributed to low advice and treatment is the inadequate integration of mental health into general PHC, and insufficient or inadequate training to capacitate health workers to provide treatment for mental illnesses. In Malawi, integration of mental health services into primary healthcare has not been fully implemented despite the mental health policy advocating for integration [32]. A study in neighboring South Africa found that lack of integration of mental health services was one of the barriers to substance use treatment in a PHC [33]. However, integrating mental health and HIV/AIDS programmes have found promising results among those who have mental health problems. For example, a study conducted in Ugandan HIV clinics found that on average 76.3% of participants were screened daily using the PHQ-2 [34]. Similar promising results were reported in a Malawian study which reported that following a capacity building programme, 88.3% and 93.2% of newly diagnosed seropositive clients were screened for depression at two PHCs [25].

Given that participants in this study reported that they visited the ART clinics relatively often, on average five times a year, this would be optimal for providing mental health services at the same time, thereby narrowing the treatment gap and reducing the occurrence of missed opportunities for treatment at the clinics.

### Recommendations

The findings of this study point to a number of recommendations to improve delivery of mental health services at ART clinics. These are to: 1) provide psychoeducation to all clinic staff, to raise awareness and destigmatize mental illnesses, 2) advocate for further rollout of mhGAP training to empower COs and nurses to screen for CMDs and alcohol use, 3) add guidelines for screening and managing CMDs and alcohol use into the guidelines for management of HIV, 4) provide brief self-screening questionnaires in waiting rooms to decrease potential screening time for nurses, 5) increase human resources and staff numbers at the clinics so that they feel they have more time to dedicate to mental health care, beyond HIV care, and lastly, 6) advocate to improve and accelerate implementation of the mental health policy in Malawi, which makes provision for integrating mental health care into primary care.

## Study limitations

The study has several limitations. First, this was a cross-sectional clinic survey and therefore causal relationships cannot be addressed, as the data represents results from a single time point. Second, this study relied on a self-reporting questionnaire and therefore was susceptible to biases such as responder bias, recall bias, and social desirability bias in the sense that participants were asked about interventions offered to them for the past 12 months, and they may have altered their answers somewhat to please the data collector. Third, the study used a version of the AUDIT that was still being validated in Malawi, although it has been validated in other LMICs. Finally, we were not able to develop regression models for the 87 participants who screened positive for any mental health problems.

## Conclusion

This study found that enquiry about CMDs and risky alcohol use by COs and nurses in three ARV clinics in Zomba was low, and that most COs and nurses working in the clinics did not provide advice or treatment to clients for CMDs or risky alcohol use. This was despite many participants wanting to receive advice or treatment for these conditions. Failure by COs and nurses to give advice or treat CMDs and risky alcohol use in ART clinics represent missed opportunities to address the mental health of people living with HIV, particularly as they visit ART clinics often. COs and nurses may be well positioned to do this, but appear to be limited by a lack of awareness, guidance, and resource capacity to do so. Providing psychoeducation for staff, guidelines for screening and managing CMDs and risky alcohol use, introducing mhGAP training, and increasing human resources may be a few ways of improving mental health service provision at ART clinics in Malawi.
